# Dynamic and features of SARS-CoV-2 infection in Gabon

**DOI:** 10.1038/s41598-021-87043-y

**Published:** 2021-05-06

**Authors:** Amandine Mveang Nzoghe, Guy-Stephan Padzys, Anicet Christel Maloupazoa Siawaya, Marisca Kandet Yattara, Marielle Leboueny, Rotimi Myrabelle Avome Houechenou, Eliode Cyrien Bongho, Cedrick Mba-Mezeme, Ofilia Mvoundza Ndjindji, Jean Claude Biteghe-Bi-Essone, Alain Boulende, Paulin N. Essone, Carene Anne Alene Ndong Sima, Ulysse Minkobame, Carinne Zang Eyi, Bénédicte Ndeboko, Alexandru Voloc, Jean-François Meye, Simon Ategbo, Joel Fleury Djoba Siawaya

**Affiliations:** 1 Unité de Recherche et Diagnostics Spécialisé, Service Laboratoire, CHU-Mère-EnfantFondation Jeanne EBORI, Libreville, Gabon; 2 Département de Biologie Cellulaire et Physiologie, Faculté Des Sciences, Université Des Sciences Et Techniques de Masuku, Franceville, Gabon; 3 Hôpital Des Instruction des Armes D’Akanda, Libreville-Nord, Gabon; 4 Laboratoire National de Santé Publique, Libreville, Gabon; 5 Pôle mère, CHU- Mère-Enfant Fondation Jeanne EBORI, Libreville, Gabon; 6 Pôle enfant, CHU- Mère-Enfant Fondation Jeanne EBORI, Libreville, Gabon; 7 Département de Biologie Cellulaire and Moléculaire-Génétique, Faculté de Médecine, Université Des Sciences de La Santé, Libreville, Gabon; 8 Division of Molecular Biology and Human Genetics, Faculty of Medicine and Health Sciences, Stellenbosch University, Stellenbosch, 7505 South Africa; 9 Centre de Recherches Médicales de Lambaréné, BP 242, Lambaréné, Gabon

**Keywords:** Disease prevention, Public health, Epidemiology, Outcomes research

## Abstract

In a context where SARS-CoV-2 population-wide testing is implemented, clinical features and antibody response in those infected have never been documented in Africa. Yet, the information provided by analyzing data from population-wide testing is critical to understand the infection dynamics and devise control strategies. We described clinical features and assessed antibody response in people screened for SARS-CoV-2 infection. We analyzed data from a cohort of 3464 people that we molecularly screened for SARS-CoV-2 infection in our routine activity. We recorded people SARS-CoV-2 diagnosis, age, gender, blood types, white blood cells (WBC), symptoms, chronic disease status and time to SARS-CoV-2 RT-PCR conversion from positive to negative. We calculated the age-based distribution of SARS-CoV-2 infection, analyzed the proportion and the spectrum of COVID-19 severity. Furthermore, in a nested sub-study, we screened 83 COVID-19 patients and 319 contact-cases for anti-SARS-CoV-2 antibodies. Males and females accounted for respectively 51% and 49% of people screened. The studied population median and mean age were both 39 years. 592 out of 3464 people (17.2%) were diagnosed with SARS-CoV-2 infection with males and females representing, respectively, 53% and 47%. The median and mean ages of SARS-CoV-2 infected subjects were 37 and 38 years respectively. The lowest rate of infection (8%) was observed in the elderly (aged > 60). The rate of SARS-Cov-2 infection in both young (18–35 years old) and middle-aged adults (36–60 years old) was around 20%. The analysis of SARS-CoV-2 infection age distribution showed that middle-aged adults accounted for 54.7% of SARS-CoV-2 positive persons, followed respectively by young adults (33.7%), children (7.7%) and elderly (3.8%). 68% (N = 402) of SARS-CoV-2 infected persons were asymptomatic, 26.3% (N = 156) had influenza-like symptoms, 2.7% (N = 16) had influenza-like symptoms associated with anosmia and ageusia, 2% (N = 11) had dyspnea and 1% (N = 7) had respiratory failure, which resulted in death. Data also showed that 12% of SARS-CoV-2 infected subjects, had chronic diseases. Hypertension, diabetes, and asthma were the top concurrent chronic diseases representing respectively 58%, 25% and 12% of recorded chronic diseases. Half of SARS-CoV-2 RT-PCR positive patients were cured within 14 days following the initiation of the anti-COVID-19 treatment protocol. 78.3% of COVID-19 patients and 55% of SARS-CoV-2 RT-PCR confirmed negative contact-cases were positive for anti-SARS-CoV-2 antibodies. Patients with severe-to-critical illness have higher leukocytes, higher neutrophils and lower lymphocyte counts contrarily to asymptomatic patients and patients with mild-to-moderate illness. Neutrophilic leukopenia was more prevalent in asymptomatic patients and patients with mild-to-moderate disease for 4 weeks after diagnosis (27.1–42.1%). In Patients with severe-to-critical illness, neutrophilic leukocytosis or neutrophilia (35.6–50%) and lymphocytopenia (20–40%) were more frequent. More than 60% of participants were blood type O. It is also important to note that infection rate was slightly higher among A and B blood types compared with type O. In this African setting, young and middle-aged adults are most likely driving community transmission of COVID-19. The rate of critical disease is relatively low. The high rate of anti-SARS-CoV-2 antibodies observed in SARS-CoV-2 RT-PCR negative contact cases suggests that subclinical infection may have been overlooked in our setting.

## Introduction

WHO declared the COVID-19 pandemic to be a Public Health Emergency of International Concern on January 30th, 2020. As the spread of the virus was expanding worldwide, the first case of COVID-19, in Africa, was reported on February 14th, and a month later on March, 13th, Gabon declared its first case. Gabon was among the few African countries that rapidly and successfully scaled up and implemented detection, prevention, and control measures. The country opted very quickly for a SARS-CoV-2 population-wide testing strategy. In this country of 2.1 million inhabitants, the population is essentially young (54.6% are under 25) (PNUD). With 60 sampling sites and a network of 15 RT-PCR laboratories located across the country, Gabon performs between 2000 and 5000 tests per day. Between March, 13th and November 13th, the country tested 266,271 people and recorded a total of 9069 SARS-CoV-2 RT-PCR positive cases (3.4% infection rate), 8938 were cured (98.5% cured rate), 65 active cases and 58 deaths (0.6% case fatality rate) (Country report).

Today, original factual researches on SARS-CoV-2 infection dynamics in Africa are scarce. We have few studies from South Africa (showing the heterogeneity of the pandemic)^1^, and Nigeria (showing an early slow epidemic trajectory, while highlighting the iceberg of asymptomatic cases in the country)^2^. Most studies on Africa are model-based, or commentaries (work of reflection on COVID-19)^3–6^.

As the COVID-19 pandemic continues, the identification of related risk factors is critical to mitigating COVID-19 detrimental impact by designing targeted interventions for people at risk. New shreds of evidence suggest that, in addition to the generally accepted factors, such as diabetes, that heightens COVID-19 related morbidity and mortality^7^, blood type may influence the course of COVID-19. Indeed, studies suggest that people with blood type O contrarily to those with blood type A, B or AB have a lower risk of COVID-19 infection and a reduced likelihood of severe disease outcomes after infection^8, 9^. If there is a true association between blood type and vulnerability to SARS-CoV-2 infection and COVID-19 progression, the determination of a population blood types distribution may help anticipate the dynamic of SARS-CoV-2 infection and the modeling of COVID-19 health impacts.

Gabon is an example of Africa's resilience to COVID-19. Analyzing and understanding the dynamic of SARS-CoV-2 infection in this unique setting may help other countries in the fight against the COVID-19 pandemic. However, we anticipate that this study will also highlight facts that may silently contribute to community transmission of COVID-19.

## Methods

The present study is based on the retrospective analysis of data from routine COVID-19 care activities. From March to August 2020, we routinely screened 3464 people for SARS-CoV-2 infection. Viral RNA extraction was fully automated. We used the ExiPrep Dx Viral DNA/RNA Kit on the ExiPrep 16 Plus instrument (Bioneer, Korea). SARS-CoV-2 diagnostic was established by real-time RT-PCR targeting the SARS-CoV-2 E and RdRp genes (AccuPower SARS-CoV-2 Real-Time RT-PCR Kit on the Exicycler 96 (Bioneer, Korea)).

Using a medical information questionnaire, we collected a range of data from the participants including gender, age, symptoms, concurrent chronic diseases and blood type group. NIH clinical spectrum of SARS-CoV-2 infection was used to classify patients. We calculated the age-based distribution of SARS-CoV-2 infection based on SARS-CoV-2 RT-PCR test results. COVID-19 positive patients followed an anti-COVID-19 treatment protocol, which consisted of oxygen therapy, hydroxychloroquine (250 mg once on day 1, followed by 125 mg daily for 10 days), azithromycin (500 mg for 5 days), vitamin C (500 mg per day for 10 days), zinc tablet (15 mg per day for 10 days) and dexchlorpheniramine (2 mg daily for 10 days). Patients were followed and regularly retested (day 8, 10, 12, 14, 16, 20, 22, 30, 40 and 50) until they had two subsequent negative tests. Based on that protocol, we established a timeframe indicative of the length of time it took our COVID-19 patients’ test results to come back negative (we called it time to the negativity). We analyzed the proportion and the spectrum of COVID-19 severity. Furthermore, we analyzed the variance of leukocytes counts according to the spectrum of COVID-19 severity. Also, we analyzed the prevalence of anti-SARS-CoV-2 antibodies (screened using both kits from Roche and bioMerieux, France) in selected patients and contact-cases.

This study was approved by the institutional review board of the Mother and Child University Hospital in Libreville on April, 27th 2020 (CHUMEJE/SARS-CoV-2/S2) and registered with the Gabonese Scientific Comity on COVID-19 on November, 16th 2020. The Gabonese COVID-19 care policy requires that all patients sign an informed consent. Additionally, in the setting of Mother and Child University Hospital, anticipating the use of collected data for epidemiological surveillance studies, we obtained informed consent from each participant at the time of diagnosis. The study was conducted according to Gabonese guidelines and regulations.

### Statistical analysis

The statistical analysis was performed using GraphPad Prism software version 8. The Mann–Whitney U-test was performed to compare two groups. When more than two groups were compared, we used the ANOVA one-way non-parametric multiple comparisons test (Kruskal–Wallis test) coupled to the Dunn’s multiple comparisons test. The threshold of significance was a p-value 0.05.

## Results

### Studied population

We analyzed data of 3464 people screened for SARS-CoV-2 infection. Males and females accounted for respectively 51% and 49% of people screened. The studied population median and mean age were both 39 years. 592 out of 3464 people (17.2%) were diagnosed with SARS-CoV-2 infection. 53% of infected individuals were males and 47% were females.

### Age-based distribution of SARS-CoV-2

3268 tested people had their age correctly informed. Subjects ages ranged from under 1 year old to 89 years old, with a median age of 39. The median and mean age of SARS-CoV-2 infected subjects were 37 and 38 years respectively. Table 1 shows the rate of infection by age groups. The lowest rate of infection (8%) was observed in the elderly (aged > 60).

**Table 1:** SARS-CoV-2 age-associated infection rate.

Age groups (in years)	SARS-CoV-2 positive	SARS-CoV-2 negative	Total	Positivity rate
0–17 (Children)	44	284	328	13%
18–35 (young adults)	193	788	981	20%
36–60 (middle age adults)	313	1374	1687	19%
60+ (elderly)	22	250	272	8%
**Total**	**572**	**2696**	**3268**	

**Figure 1:**
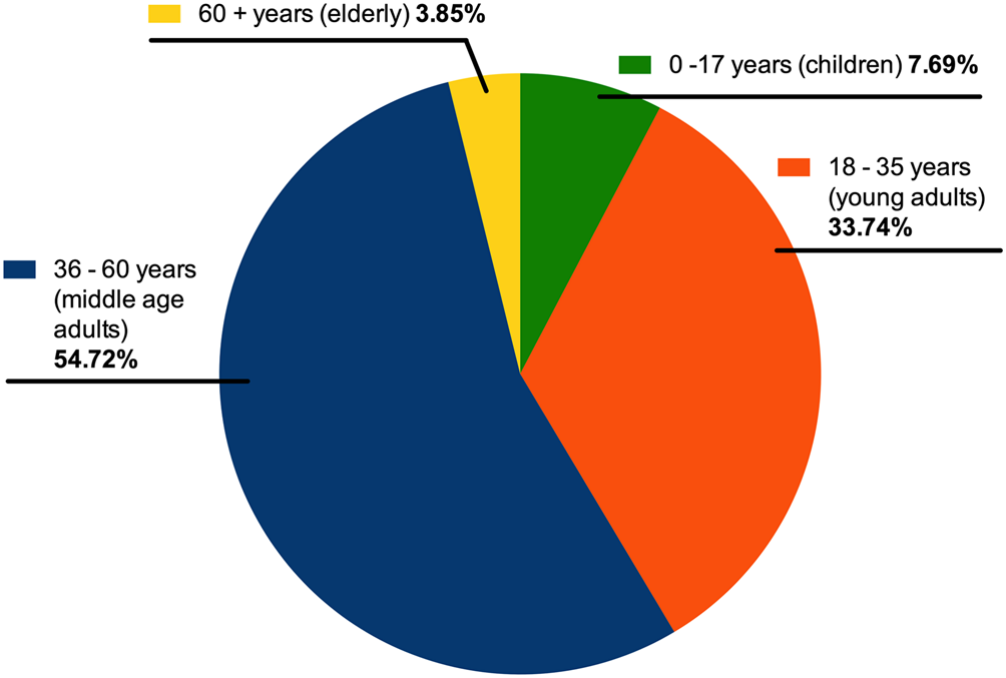
Age distribution of SARS-CoV-2 RT-PCR test. Middle-age adults represented 54.72% of SARS-CoV-2 positive persons, young adults 33.74%, children 7.69% and elderly 3.85%.

The rate of SARS-Cov-2 infection in both young (18–35 years old) and middle-aged adults (36–60 years old) was around 20%. The analysis of SARS-CoV-2 infection age distribution showed that middle-aged adults accounted for 54.7% of SARS-CoV-2 positive persons, followed respectively by young adults (33.7%), children (7.7%) and elderly (3.8%) (Fig. 1).

### Time to SARS-CoV-2 negativity

53% of SARS-CoV-19 PCR positive patients were cured (virus-free) within 14 days following the initiation of medical care (Table 2). Subsequently, 31%, 8% and 8% were cured respectively 20, 30, 40 and 50 days after the initiation of treatment.

**Table 2:** Time to SARS-CoV-2 RT-PCR conversion from positive to negative.

	Day 8	Day 10	Day 14	Day 20	Day 30	Day 40 +
N (%)	36 (17%)	40 (19%)	36 (17%)	64 (31%)	17 (8%)	16 (8%)
Mean	44.4	34.2	38.4	39.7	45.2	43.4
Median	42	34	38.5	37.0	43	39.5
Minimum	13	9	8	9	18	28
Maximum	73	57	73	73	66	66
Standard deviation	14.1	13.3	13.7	11.8	13.0	11.9
25th percentile	34	26	31.8	31.1	39	36
50th percentile	42	34	38.5	37	39	39.5
75th percentile	55	44	44	45.5	52	45.8

### Symptomatology of SARS-CoV-2 positive subjects

Out of 592 SARS-CoV-2 infected persons, 402 (68%) persons were asymptomatic, 156 (26.3%) had influenza-like symptoms, 16 (2.7%) had influenza-like symptoms associated with anosmia and ageusia (mild to moderate illness), 11 (2%) patients had dyspnea (severe illness) and 7 (1%) patients had respiratory failure resulting in death (critical illness). Summarily, among symptomatic cases, 90% had mild symptoms, 6% were severe cases, and 4% were critical cases.

### Chronic diseases among SARS-CoV-2 positive subjects

**Figure 2:**
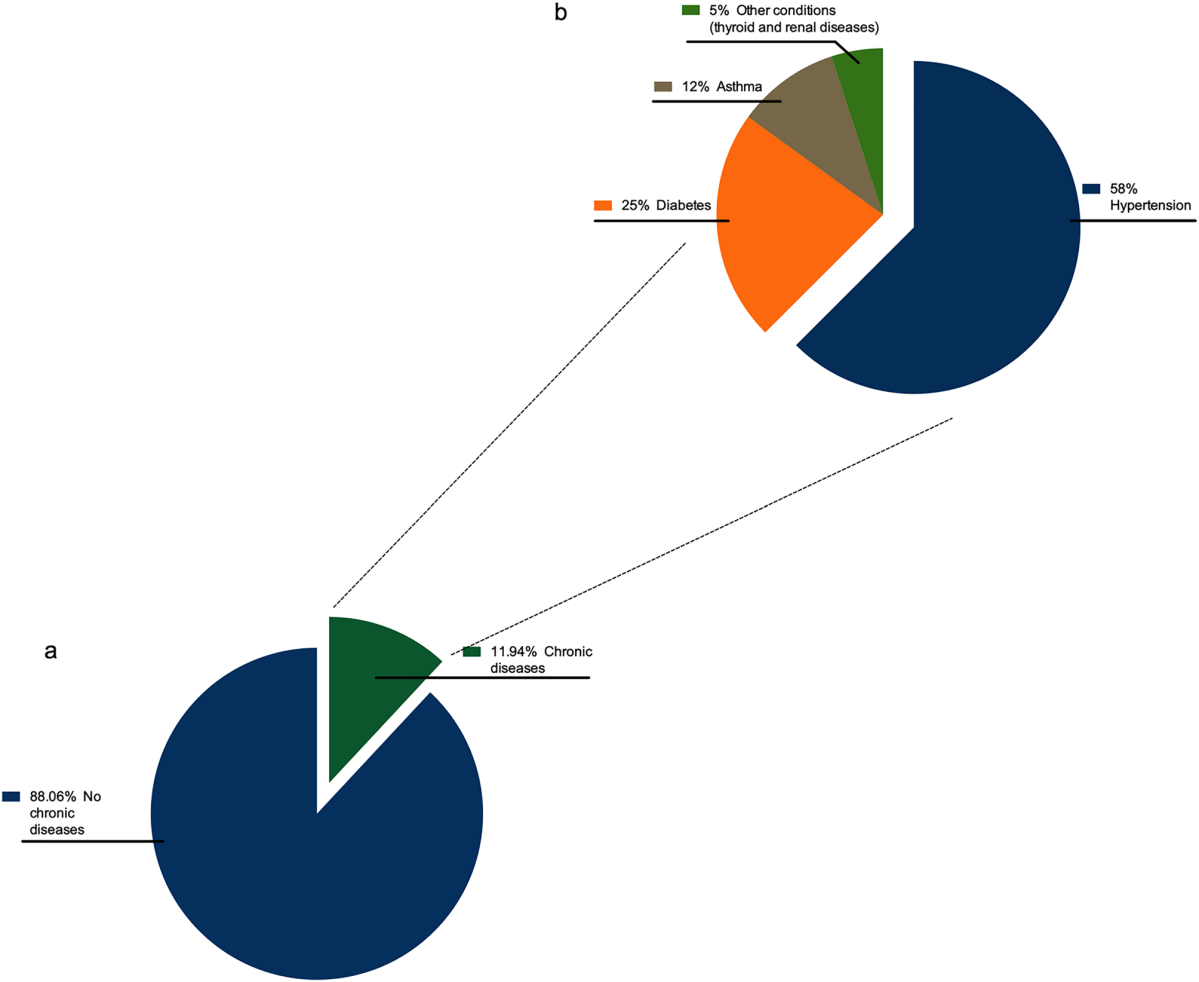
Chronic diseases among SARS-CoV-2 positive subjects. (**a**) 12% of SARS-CoV-2 infected subjects, had chronic diseases. (**b**) Hypertension represented 58% of recorded chronic diseases, followed respectively by diabetes (25%) and asthma (12%). Other conditions (including thyroid and renal diseases) represented 5% of recorded chronic disease cases.

12% of SARS-CoV-2 infected subjects had chronic diseases (Fig. 2a). Respectively, 7%, 3% and 1.5% of SARS-CoV-2 infected subjects had hypertension, diabetes (associated or not with hypertension) and asthma. Hypertension represented 58% of recorded chronic disease cases, followed respectively by diabetes (25%) and asthma (12%). Other conditions (including thyroid and renal diseases) represented 5% of recorded chronic disease (Fig. 2b).

### Analysis of selected formed blood elements based on COVID-19 spectrum between diagnosis and week 4

#### Leukocytes

**Figure 3:**
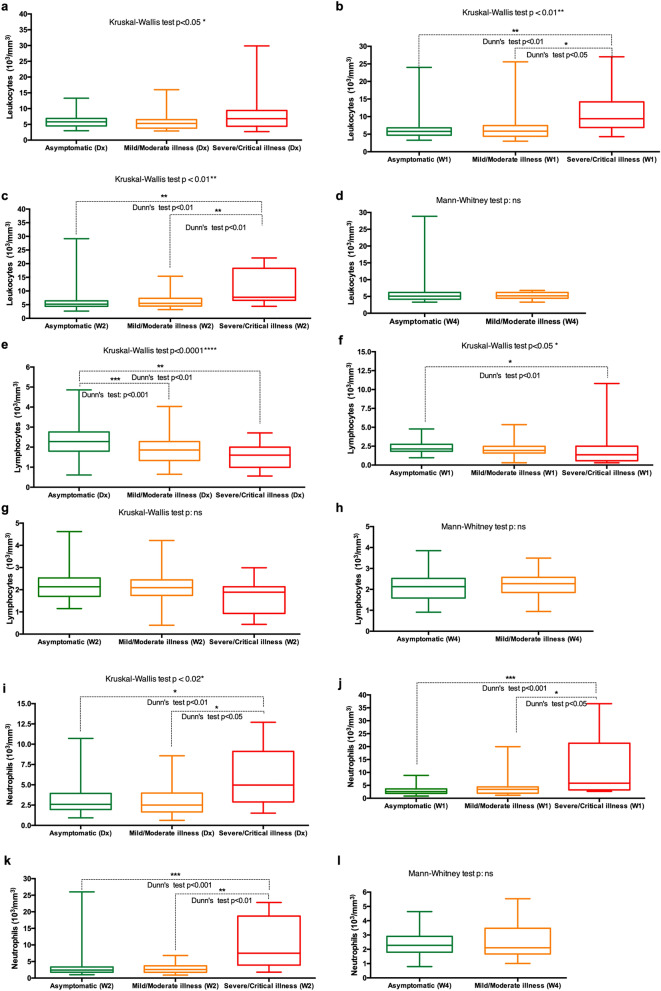
(**a**–**l**) Temporal changes in the count of leukocytes (**a**–**d**), lymphocytes (**e**–**h**) and neutrophils (**i**–**l**) in patients with different COVID-19 severity spectrums. Blood samples were collected and analyzed at diagnosis (Dx), week 1 (W1), week 2 (W2) and week 4 (W4).

Leukocytes’ analysis of variance showed that absolute leukocyte count differs significantly depending on the spectrum of COVID-19 severity (p < 0.05) (Fig. 3). The differences are more evident at week 1 and 2 following diagnosis. Both at week 1 and week 2 patients with severe or critical illness had statistically significantly higher leukocyte counts than asymptomatic patients and patients with mild or moderate illness (p < [0.01–0.05]) (Fig. 3b,c). The analysis of leukocyte counts base on clinical interpretation showed that 1.4–4% of asymptomatic patients had leukocytosis and 30–38% had leukopenia. In patients with mild-to-moderate illness, the rate of leukocytosis ranged between 3 and 5.7%, whereas 41.2–44% had leukopenia. In severe-to-critically ill patients, the rate of leukocytosis ranged between 18.5–41.6%. The rate of leukopenia ranged between 8.3 and 25% (Table 3).

**Table 3:** Characteristics of differential white blood cell counts in different severity groups of COVID-19.

		Day1	Week 1	Week 2	Week 14	Day 1	Week 1	Week 2	Week 14	Day 1	Week 1	Week 2	Week 14
Leukocytes (Reference range: 4000–10 000/mm^3^)	Leukocytosis	2%	1.1%	4%	0%	4.3%	3%	5.7%	5.5%	18.5%	36.4%	41.6%	A very low number of subjects
	Leukopenia	33%	30%	38%	46%	43.5%	41.2%	47%	44%	25%	9%	8.3%	A very low number of subjects
Lymphocytes (Reference range: 1000–4500/mm^3^)	Lymphocytosis	0%	0%	0%	0%	0%	3%	0%	0%	0%	4.6%	0%	
	Lymphocytopenia	1.3%	1.1%	0%	3.4%	13%	5.2%	5%	4.8%	21.4%	40%	20%	A very low number of subjects
Neutrophils (Reference range: 2000–7500/mm^3^)	Neutrophilia	0.6%	4.1%	2.4%	0%	7.7%	13.5%	6.1%	0%	35.7%	45.4%	50%	
	Neutropenia	27.1%	29%	35%	29%	33.8%	24.3%	30.3%	42.1%	14.3%	0%	0%	A very low number of subjects
Monocytes (Reference range: 200–1000/mm^3^)	Monocytosis	3.5%	2.8%	3.5%	5.1%	10.6%	10.7%	11%	6.2%	13.3%	9%	18.1%	
	Monocytopenia	2.8%	4.2%	3.5%	2.5%	0%	4.2%	5.7%	0%	0%	0%	18.11%	A very low number of subjects
Thrombocytes (Reference range: 150 000–400 000/mm^3^)	Thrombocytosis	0%	1.3%	0%	0%	1.5%	2.8%	0%	0%	0%	0%	0%	
	Thrombocytopenia	16.9%	6.5%	7.9%	5%	19.4%	11.4%	5.9%	6%	20%	0%	25%	A very low number of subjects

#### Lymphocytes

The initial lymphocytes absolute count was significantly higher in asymptomatic patients than in both mild-to-moderate and severe-to-critical COVID-19 patients (p < [0.001–0.01]) (Fig. 3e). At week 1, only the difference between asymptomatic patients and patients with mild-to-moderate illness remained significantly different (p < 0.01) (Fig. 3f). 1.1 to 3.4% of asymptomatic patients had lymphocytopenia. No lymphocytosis was observed in asymptomatic patients. For patients with mild-to-moderate, the rate of lymphocytopenia ranged between 4.8% and 13%. 3% of patients with mild-to-moderate illness developed a lymphocytosis (between diagnosis and week 4) (Table 3).

#### Neutrophils

From diagnosis, week 1 and week 2 after, patients with severe-to-critical illness had their absolute neutrophils count significantly higher than both asymptomatic patients and patients with mild-to-moderate illness (p < [0.001–0.05]) (Fig. 3i–k). The rate of neutrophilia ranged from 0.6 to 4.1% for asymptomatic patients, whereas the rate of neutropenia ranged between 27.1 to 35%. In patients with mild-to-moderate, the rate of neutrophilia ranged between 6.1 and 13.5%. 24.3 to 42.1% of patients with mild-to-moderate illness developed neutropenia. The rate of neutrophilia ranged between 35.7 and 50% for patients with severe-to-critical illness. The rate of neutropenia ranged between 0 and 14.3% (Table 3).

#### Monocytes

No significant differences in the absolute count of monocytes were observed between the patients’ groups. Respectively, the rate of monocytosis and monocytopenia did not exceed 3.5% and 4.2% for asymptomatic patients. For patients with mild-to-moderate illness, the rate of monocytosis ranged between 6.2 and 11%. Monocytopenia rate did not exceed 5.7% and in patients with mild-to-moderate illness (Table 3).

#### Thrombocytes

**Figure 4:**
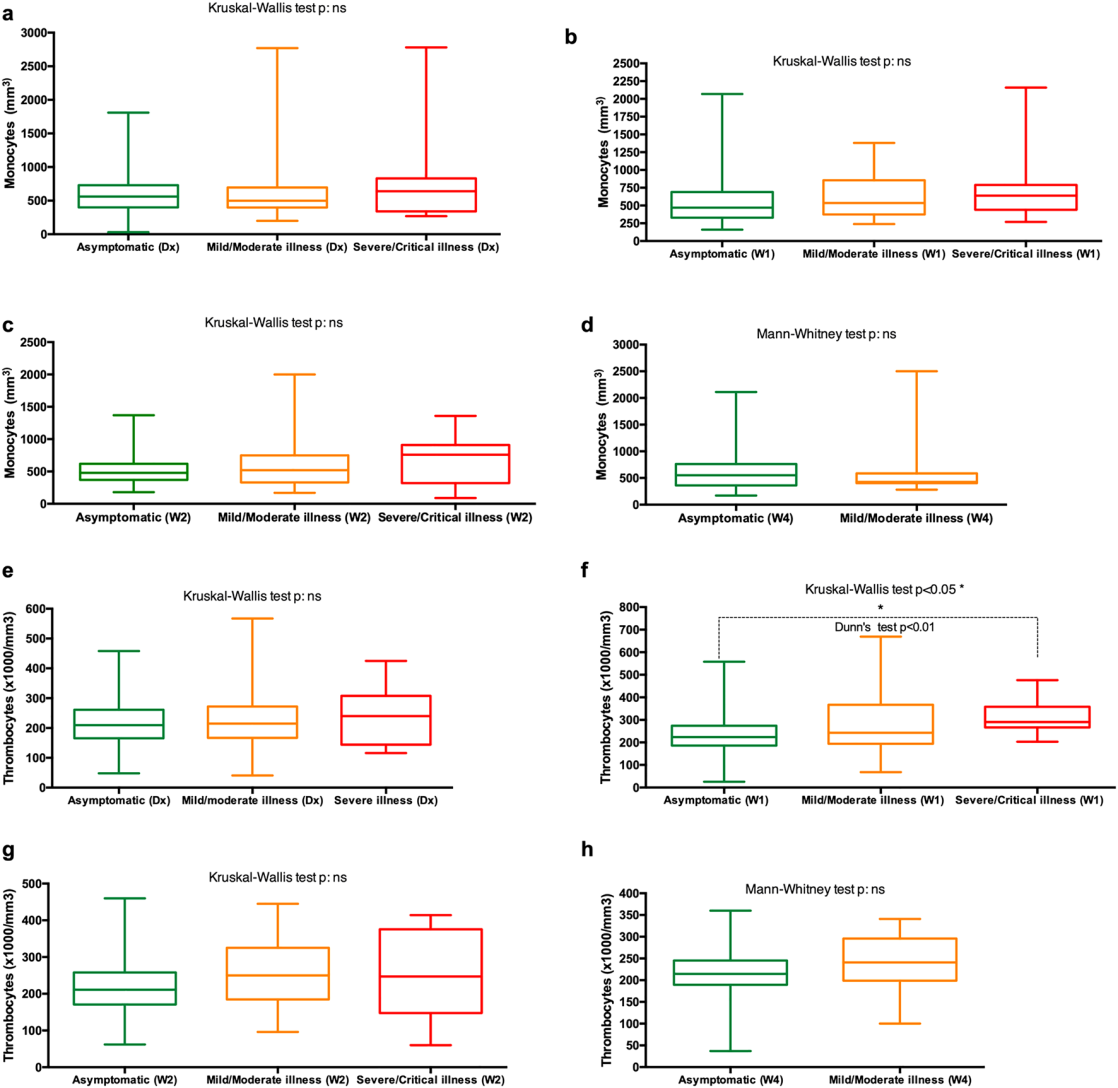
(**a**–**h**) Temporal changes in the count of monocytes (**a**–**d**) and thrombocytes (**e**–**h**) in patients with different COVID-19 severity spectrums. Blood samples were collected and analyzed at diagnosis (Dx), week 1 (W1), week 2 (W2) and week 4 (W4).

Data analysis showed that at week 1 after the initial diagnosis, patients with severe-to-critical illness had a significantly higher absolute count of thrombocytes than asymptomatic patients. No other significant differences were observed (Fig. 4). The clinical interpretation of data revealed that the rate of thrombocytosis did not exceed 1.3% and 2.8% for asymptomatic patients and patients with mild-to-moderate illness respectively. Thrombocytopenia was prevalent in all patients. Its rate ranged between 6.5 and 16.9% for asymptomatic patients and between 5.9 and 19.4% for patients with mild-to-moderate illness. In patients with severe-to-critical illness, the rate of thrombocytopenia ranged between 6 and 25% (Table 3).

### Anti-SARS-CoV-2 antibodies among patients and contact-cases

**Figure 5:**
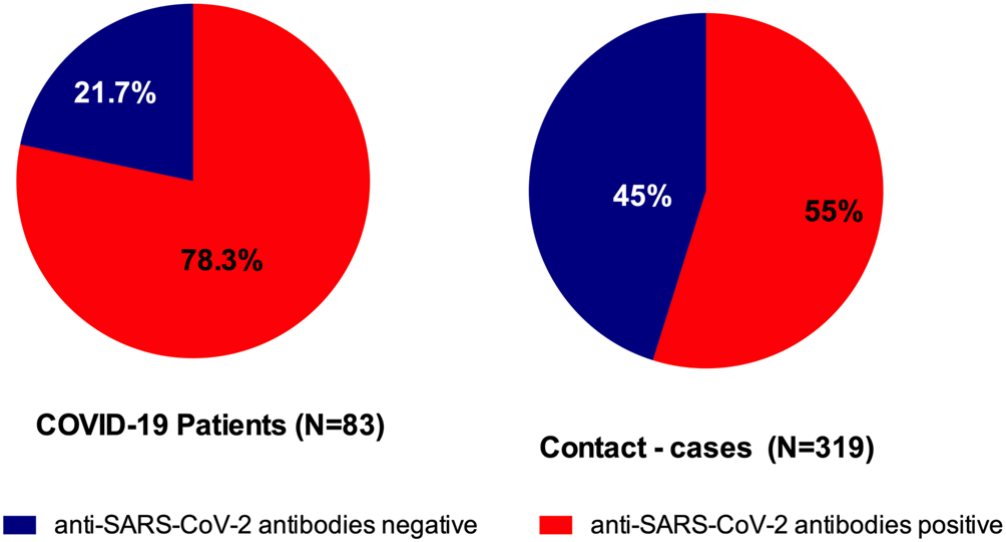
Anti-SARS-CoV-2 antibodies among patients and contact-cases. 78.3% of COVID-19 patients and 55% of SARS-CoV-2 PCR negative contact-cases had anti-SARS-CoV-2 antibodies.

83 COVID-19 patients and 319 contact-cases were screened for anti-SARS-CoV-2 antibodies among which 78.3% of COVID-19 patients developed anti-SARS-CoV-2 antibodies and 55% of SARS-CoV-2 PCR negative contact-cases had anti-SARS-CoV-2 antibodies (Fig. 5).

### SARS-CoV-2 infection and blood groups A, B, AB and O

A total of 1159 subjects informed their blood group. Respectively, 60.3%, 21.1%, 16.9% and 2.7% of subjects were O, A, B and AB. The SARS-CoV-2 infection rate was higher among A (11.1%) and B (12.4%) blood group type and lower among AB group type (6.6%), compared with group type O (10.7%).

Looking at ABO, Rh groups Respectively, 59%, 20%, 15% and 2.6% of subjects were O^**+**^, A^**+**^, B^**+**^ and AB^**+**^. O^**−**^, A^**−**^, B^**−**^ represented respectively 1.3%, 1.1%, and 0.9% of subjects only 1 (~ 0.01%) subject was AB^**−**^. The rate of SARS-CoV-2 infection was 10% in both people of the blood group O^**+**^ and A^**+**^. In people of the blood group B^**+**^**,** the rate of infection was 13%. The infection rate in people of the blood group O^**−**^ and A^**−**^ were respectively 20%* and 23%* (*very small number of people of these blood groups) (Table 4). No positive was recorded among the 10 subjects of the blood group B^**-**^. Only 1 subject was AB^-^ and he was SARS-CoV-2 RT-PCR negative.

**Table 4:** Infection rate by blood groups ABO and Rh (D).

Blood groups	A		B		AB		O	
SARS-CoV-2 positive	27		23		2		75	
SARS-CoV-2 negative	215		162		29		626	
Total	242		185		31		701	
SARS-CoV-2 Infection rate	11.1%		12.4%		6.4%*		10.7%	

Blood groups and Rh(D)	A−	A +	B−	B +	AB−	AB +	O−	O +
SARS-CoV-2 positive	3	24	0	23	0	2	3	72
SARS-CoV-2 Negative	10	205	10	152	1	28	12	614
Total	13	229	10	175	1	30	15	686
SARS-CoV-2 Infection rate	23%*	10%	0%*	13%	0%*	7%*	20%*	10%

Rh (D)	Rh +				Rh-			
SARS-CoV-2 Positive	98				6			
SARS-CoV-2 Negative	1022				33			
Total	1120				39			
SARS-CoV-2 Infection rate	8.75%				15.4%*			

*A very low number of subjects to draw a meaningful interpretation.

## Discussions

Africa's resilience to COVID-19 is often attributed based on deductive reasoning to the youth of its population. This study is one of the first describing SARS-CoV-2 infection in an African context. The study looked at aged-stratified infection rate, symptomatology, the prevalence of chronic diseases, time to SARS-CoV-2 negativity (from diagnosis) and the development of anti-SARS-CoV-2 immunity.

Data showed that in our setting, from March to August 2020, the SARS-CoV-2 positive rate based on RT-PCR tests was 17.2%. SARS-CoV-2 age distribution analysis showed that young and middle-aged adults accounted for 88.4% of SARS-CoV-2 positive persons, followed respectively by children (7.7%) and elderly (3.8%). Young and middle-aged adults likely contribute to community transmission of COVID-19 in our setting. The lowest rate of infection (8%) was observed in the elderly and children (13%), whereas the highest infection rate was observed in young and middle-aged adults (~ 19%). These figures can be explained by the age stratification in our context (Gabonese context), where elderly, children and young/middle-aged adults represent about 4%, 40% and 56% of the population respectively. Other explanations include population activity (elderlies are less active) and the country's strong measures to protect the most vulnerable segment of the population (early total confinement, obligation to wear a mask in public places, cases close contact cases quarantine, etc.). The median age of SARS-CoV-2 positive subjects in our setting was 39 years, which is lower than what was observed respectively in France (44 years)^10^. Moreover, in the western world elderlies represented between 17 and 40% of COVID-19 cases^11^. Today, there is a change in the trend of COVID-19 in the west^12, 13^. The gap between age groups shrank considerably^11, 13^. Like in Africa, young and middle-aged adults are becoming the cornerstone of community transmission of COVID-19.

Symptomatology analysis in this African context, where SARS-CoV-2 population-wide testing is implemented, revealed that nearly 70% of SARS-CoV-2 positive subjects are asymptomatic. 29% showed mild to moderate symptoms (influenza-like symptoms associated with anosmia and ageusia) and only 2% presented severe symptoms (mild dyspnea) and 1% showed critical symptoms (respiratory failure) and died. The selected features of SARS-CoV-2 in this African context are in some aspects similar but in others different to what was observed elsewhere. Except for the report from the Tuscany region of Italy (50–75% of asymptomatic cases)^14^, the proportion of SARS-CoV-2 positive asymptomatic cases in our context is different from what is observed in developed countries (approximately 40% to 45% of asymptomatic cases^11, 13^. Also, looking at the spectrum of disease severity in symptomatic patients, our figures are not that much different from what was observed in China^15^, or France^16^ (80–90% of mild cases). The level of comorbidities in SARS-CoV-2 infected persons was relatively low in our setting. Only 3% of SARS-CoV-2 infected persons had diabetes, the number one factor that heightens COVID-19 related morbidity and mortality^7^. Gabon is both an example of Africa's resilience to COVID-19 and an illustration that the early detection of asymptomatic cases strategy allows efficient control of the pandemic.

Our analysis of white blood cells (leukocytes) feature of COVID-19 patients revealed that patients with severe-to-critical illness have higher leukocytes, higher neutrophils and lower lymphocyte counts contrarily to asymptomatic patients and patients with mild-to-moderate illness. Very similar features were observed in Chinese COVID-19 patients^17, 18^. Neutrophilic leukopenia was more common in asymptomatic patients and patients with mild-to-moderate disease for 4 weeks after diagnosis (27.1–42.1%). In Patients with severe-to-critical illness, neutrophilic leukocytosis or neutrophilia (35.6–50%) and lymphocytopenia (20–40%) were more prevalent. In the literature, neutrophilia and lymphocytopenia have been reported as frequent features of in patients with severe-to critical illness^18, 19^. Also, thrombocytopenia was a feature observed in all patients’ groups with the highest rate around the first week after diagnosis (11.4–25%). A published meta-analysis study by *Muhammad S. Asghar *et al.^20^ shows thrombocytopenia as a significant feature of COVID-19 patients. Overall, the whites blood cells and thrombocytes features of African COVID-19 patients are similar to what was observed in other populations.

With nearly 80% of COVID-19 patients producing anti-SARS-CoV-2 antibodies, our data indicates that Africans antibody response following SARS -CoV-2 infection seems to be similar to what is observed in Europe^21^ and Asia^22, 23^. However, the durability of this acquired immunity still needs to be investigated in the African context to establish the value of antibody-based herd immunity and perhaps validate the concept of “immunity passports”. The high rate of anti-SARS-CoV-2 antibodies observed in SARS-CoV-2 PCR negative contact cases suggests that subclinical infection may have been overlooked^24^, as it is common in respiratory viruses, including SARS-CoV-2 infection^25^. It is therefore clear that case counts based on RT-PCR tests may underestimate the actual SARS-CoV-2 attack rate^24^.

More than 60% of participants, for whom the blood type was recorded, were of the blood type O. This observation is in line with the country statistics showing that more than 60% of the Gabonese population is of the blood type O (countries data). As published data suggest that people with the blood type O may have the lowest risk of SARS-CoV-2 infection and develop less severe COVID-19 disease than others^8, 9, 26^, the predominance of people with the blood type O in the population may be an additional factor explaining the low rate of COVID-19 severe disease in our setting. The SARS-CoV-2 infection rate was higher among A and B blood types compared with type O (the gap in SARS-CoV-2 infection rate between the groups did not reach 5% probably because of the relatively low numbers of people of the blood types A, B and AB. Nevertheless, our finding regarding infection prevalence was similar to what was observed by Zietz et al.^27^. A great part of the population was positive for Rh (D). Therefore, the very small number of people negative for Rh (D) made it difficult to give sense to the proportions of SARS-CoV-2 cases observed in Rh (D)-negative people.

## Conclusion

In Africa, young and middle-aged adults constitute a pool for SARs-CoV-2 and are most likely to fuel community transmission of COVID-19. Particularities to young and middle-aged adults and the eventuality of subclinical infection should be considered when designing control measures. Africans should not be complacent and should not lower their level of vigilance. Also, population-based serological studies are needed in the African context to provide insights on SARS-CoV-2 transmission and the immunological state of the population.

## Data Availability

Our data are accessible to researchers upon request. For data, sharing contact the corresponding author.
